# Digitalization of the Logistics Process in Short Food Supply Chains. An online Viable System Model application during the COVID-19 pandemic

**DOI:** 10.1007/s11213-022-09619-7

**Published:** 2022-11-02

**Authors:** Eliseo Vilalta-Perdomo, David E. Salinas-Navarro, Rosario Michel-Villarreal, Rocío García Bustamante

**Affiliations:** 1 Operations and Information Management Department, Aston University, Birmingham, UK; 2 School of Agriculture, Food and Environment, Royal Agricultural University, Cirencester, UK; 3 Universidad Iberoamericana, Puebla, Mexico

**Keywords:** COVID-19, Farmers’ markets, Group facilitation, Online Systems Thinking, Short food supply chains

## Abstract

This paper reports an ongoing exercise concerning the design of a logistics App to support operations within Farmers’ Markets in Mexico. This exercise is part of a wider research agenda focused on ‘Supporting Alternative Food Networks’ (SAFeNET). This is a research agenda to conceive, build, implement, and develop better-informing decision-making processes that support effective and efficient AFNs (also known as Short Food Supply Chains) logistics operations in a digital environment, through smooth flows of goods and information among producers, AFNs coordinators, and consumers. This view calls for taking a systemic approach to help collectives of people to improve their autonomy and viability. Initial plans were to conduct this collaborative design exercise, using the Viable System Model (VSM) as a conversational tool. Accordingly, a series of face-to-face interviews and a focus group were planned. However, the lockdown due to COVID-19 forced researchers to abandon the face-to-face option and conduct the primary data collection online. The VSM intervention had to be adapted for its use on an online platform, in such a way that the platform would support knowledge building interactively, with a series of participants. This paper describes the format and visual appearance of the online VSM framework, its application, and the lessons learned through this exercise. Two points deserve to be highlighted: First, although the exercise outcome was very valuable for the next stage of the design, the participants’ capacity for collective and individual reflection during the workshop was limited. Second, participants continued adding comments via the adopted online visual collaboration platform after the workshop ended, showing an understanding of the process and commitment beyond the researchers’ expectations. The outcomes from this experiment are promissory, suggesting that online Systems Thinking interventions deserve further development.

## Introduction

This paper reports the approach followed and supportive materials developed to implement the Viable System Model (Beer 1985) online, as a conversational tool to support the improvement of collective logistics efforts of people in Short Food Supply Chains (SFSCs) during the COVID-19 pandemic. Adjustments were conceived, designed, and executed under the umbrella of a wider research agenda focused on Supporting Alternative Food Networks (SAFeNET). SAFeNET is a multidisciplinary and multinational research agenda to conceive, build, implement, and develop better-informing decision-making processes that support effective and efficient AFNs logistics operations, through smooth flows of goods and information among producers, AFNs coordinators, and consumers. In this paper, AFNs and SFSCs are used interchangeably.

Recent evidence suggests that the ongoing COVID-19 pandemic and associated lockdowns have caused significant disruption to SFSCs, impacting their sustainability performance and resilience. For instance, Farrell et al. (2020) identified that the forced closure of farmers’ markets during the COVID-19 pandemic had a negative impact on diverse sustainability aspects, including food waste and farmers’ livelihoods. Similarly, Benedek et al. (2021) found that the COVID-19 crisis was detrimental to the sustainability of SFSCs farmers in Hungary. The key to enhanced resilience for many SFSCs was the strategic reconstruction of marketing channels through digitalization, which required profound modifications of the supply chain, including logistics processes. In this sense, Michel-Villarreal et al. (2021) suggest that some SFSCs in Mexico were able to change their mode of operation by adopting online business models and home delivery during the COVID-19 pandemic, as a response to a national lockdown. This required the adoption of digital technologies such as WhatsApp and social media. However, the organizational, logistical, and technological changes required for the digitalisation of SFSCs, as well as the associated challenges, have not been documented yet. To address this, the authors proposed a research pilot to diagnose SFSCs current organizational, logistical, and technological processes for collective selling, and to determine SFSCs informational needs associated with such processes. For this purpose, primary data was collected through interviews with key actors, and a focus group was held with five SFSCs from three different states in Mexico.

To conduct this research pilot, two assumptions were considered: (a) to bring together a variety of stakeholders who are directly or indirectly involved in the processes of delivering food to the final consumer (from farm to fork), and (b) to adopt a Systems Thinking approach to study SFSCs actors and their interactions. Due to its focus on mapping control and monitoring channels within organizations, the Viable System Model (VSM) was used as a machine to generate questions (Salinas-Navarro 2003; Vilalta-Perdomo 2005), that to help collecting observations through interviews and a focus group with the stakeholders. This approach turns the VSM into a conversational tool to support stakeholders’ self-awareness and learning about their collective efforts, going beyond the common representational use of the model. However, conducting such research under the conditions of the COVID-19 pandemic was not a trivial task, particularly in the case of participatory research (Hall et al. 2021). Face-to-face interactions were not possible, due to travel restrictions and social distancing measures introduced to contain the spread of COVID-19. Thus, this paper describes an example of how to develop a web-based Systems Thinking tool based on the VSM to run an online synchronous focus group. The development, use, and test of an online VSM tool became a relevant exercise itself, as it provided an alternative way of conducting systemic interventions in cases where face-to-face interactions are not possible. This paper discusses this exercise in detail.

The structure of this paper is as follows. The first section sets the context of the two main constructs used in this text: Short Food Supply Chains (SFSCs) and the Viable System Model (VSM). The second section explains the design of the research pilot, considering some of the requirements for an online mapping exercise on SFSCs, the graphic description of VSM using the Miro© platform, and the data collection and analysis. The third section provides a series of preliminary findings concerning SFSCs and the pertinence of online VSM. Finally, the last section concludes this paper, reflecting on the impacts of using online VSM in practice and research.

## Setting the Context

As indicated above, this paper reports a series of adjustments conceived, designed, and executed to conduct an online VSM intervention, to map and support the improvement of collective logistical efforts of (digitalized) short food supply chains (SFSCs) during the COVID-19 pandemic. The first step was diagnosing SFSCs current organizational, logistical, and technological processes for collective selling, and to determine the informational needs associated with such processes. For this purpose and considering the research challenges derived from the COVID-19 pandemic, we established a course of action that consisted of applying online VSM for the mapping of digital SFSCs. Accordingly, two constructs need to be introduced; first, the notion of Short Food Supply Chains (SFSCs), second, what is the Viable System Model (Beer 1985).

### Short Food Supply Chains (SFSCs)

Late global developments such as the COVID-19 pandemic suggest that food systems are more resilient than expected (Economist 2020), but tensions on Food Supply Chains (FSCs) are far from being solved (Foroohar 2021). Many FSCs are engaged in a process of reinvention to increase their ability to anticipate and get ready for unexpected turbulences (Michel-Villarreal et al. 2021). Visibility and collaboration seem fundamental to succeed in this endeavor (Zouari et al. 2021). This paper refers to SAFeNET, a research agenda that explores one of the possible routes to increase visibility and collaboration to improve FSC resilience, by shortening the chain (Chiffoleau and Dourian 2020) and using online platforms (Shveda 2020).

‘Short Food Supply Chain’ (SFSC) is a concept popularized by Marsden et al. (2000) and later Renting et al. (2003) that focuses on the benefits expected from reducing intermediation and embedding information into food products. Some of SFSCs expected gains are “economic benefits to both producers and consumers, strengthening social relations, preserving the environment, improving nutritional aspects, and enhancing local development” (United Nations Industrial Development Organization, 2020, p.3). Building on Christopher’s (2016) definition of supply chains, Michel-Villarreal et al. (2021) interpret SFSCs as:

“Networks of connected and interdependent actors mutually and cooperatively working together to control, manage and improve the flows of information-embedded products, services, resources, and/or information, from farm to fork, seeking a reduction of intermediaries (minimal or ideally nil) and physical distance between producers and consumers”.

In this context, this paper explores SFSCs as food systems where: (a) there are none or very few intermediaries, (b) information is embedded into the food product, and (c) the physical distance between farm and fork is minimal. Furthermore, as research on SFSCs in the Global South has been neglected (Michel-Villarreal et al. 2019), we focused the research on SFSCs in a country (Mexico) where this phenomenon is incipient and remains insufficiently researched.

To investigate the organizational, logistical, and technological processes of SFSCs, we focused on the flows of communication and their control between the different subsystems that constitute SFSCs. For this purpose, we decided to make use of principles derived from cybernetics: “the science of communication and control” (Wiener 1954; Ashby 1956/1999, Beer 1975), and in particular from the Viable System Model (Beer 1985).

### Viable System Model (VSM)

The *Viable System Model* (VSM) was conceived by Stafford Beer, through a series of publications over two decades (1966, 1972, 1979, 1985). Beer’s research interest was focused on how to steer organizations towards a desired goal and behavior. For this purpose, he proposed using a model of a controller and illustrated the concept by means of a metaphor of the nervous central system (Beer 1972).

The name for this model captures two key ideas. First, the notion of ‘system’, which according to Beer (1966) is a construct used to describe sets of elements arranged in such a way that are conducive to fulfilling a purpose (‘structure’), through desirable patterns presented in specific relationships (‘organization’). Second is the concept of ‘viability’, a construct relevant when exploring ways to maintain the existence of collectives through time. In the VSM context, viability is then the system’s ability to become responsive against external disturbances from its medium (Espejo 1993). Therefore, a viable system is then “a system that is self-sustaining, or survival worthy” (Beer 2002, p.215). In the intersection of both constructs – *system* and *viability* – we may find organizations able to maintain themselves in the future, by performing effective actions, such as problem-solving or decision-making (Espejo et al. 1996).

The rationale behind the selection of VSM to map SFSCs is built in a couple of assumptions. First, viable organizations arise when “people find successful strategies for working together, to the extent that they are able to develop and maintain a group identity in spite of environmental disturbances” (Espejo et al. 1999, p. 662). Second, a prerequisite for such a system’s viability is the development of its maximum autonomy and also among its parts, without breaking its whole integrity (Beer 1993). The main benefit of using VSM is that it allows studying systems’ viability by collecting observations around five ‘systemic functions’ (Beer 1979, 1985). First, observations focused on activities of the system, to gain some understanding of the system’s behavior. The summary of such observations is named ‘implementation’ or ‘System One’. Second, observations centered on how actors negotiate conflicts between themselves, to avoid oscillations in the system’s behavior. This systemic function is named ‘co-ordination’ or ‘System Two’. Third, observations collected on how internal stability (cohesion) is achieved, the day-to-day management. This is called ‘control’ or ‘System Three’. Also relevant to this systemic function are mechanisms used to observe divergences between what is expected and what is happening. This function is known as ‘audit’ or ‘System Three Star’. Fourth, observations gathered to understand how actors in the system under study “visualize alternative futures, and invent them” (Beer 1979:243). This is called ‘intelligence’ or ‘System Four’. Finally, observations collected concerning the sense of purpose of the system and those to monitor the relation between Systems Three and Four – between the ‘theory in use’ and the ‘espoused theory’ (Argyris and Schön 1996; Espejo et al. 1996). This is known as ‘policy making’ or ‘System Five’.

Additionally, the VSM links the five systemic functions through six vertical channels:

- Intervention. This managerial channel is used to show constraints established by the ‘owners’ of the system. It describes expectations enacted by the systemic function of policymaking (System Five).
- Resource bargain. This managerial channel makes explicit which are the managerial constraints to actors’ interactions, for instance, budget negotiations.
- Accountability. This managerial channel refers to explicit commitments made by system’s actors after resource bargaining. It is usually linked to uncovering and describing aspects such as trust or participation.
- Operational axis. This operational channel concerns functional relations between the different primary activities. It is used to describe activities in terms of processes.
- Co-ordination. This channel refers to ways actors negotiate each primary activity in which they are involved, for instance, schedules, production orders, and resource allocations.
- Audit. This channel is used to explicit if the purpose ascribed to the system under observation is fulfilled or not. This is an integral and intrinsic channel, integral because the controller is part of the system under control, and intrinsic because the process of control controls the controller (Beer 1972).

**Fig. 1:**
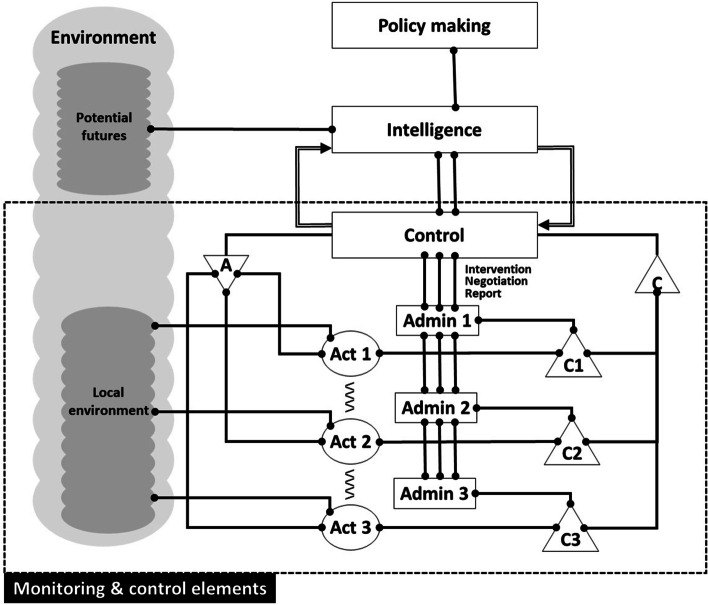
The viable system model – Focus on the monitoring & control functions

All these elements can be seen graphically in Fig. 1.

The expected benefit from using VSM to map SFSCs is the opportunity to study collective action, where an effective co-ordination of activities among members of a collective is of paramount importance. Mainstream literature on supply chains follows the managerial view that effective multi-actor coordination demands the alignment of aims and objectives (Hingley 2005; Christopher 2005). However, SFSCs are usually constituted by a myriad of members with diverse sources of motivation or interests, some of them with potentially conflicting aims and purposes, and complementary capabilities or resources that bring them together; therefore, a different approach needs to be envisioned (Hingley and Vilalta-Perdomo 2017). The VSM provides a structure to research and/or design coordination mechanisms among actors to achieve a collective identity and organization within a system. The next section shows how this can be done.

## Designing the Research Approach: Mapping SFSC with VSM

Traditional mapping tools for VSM were provided by Beer (1985). Even though these tools are usually rated as ‘cumbersome’ (Hoverstadt and Bowling 2002), their use provides useful insights on how organizations communicate to monitor and control their operations and adapt to external disturbances (Salinas-Navarro 2003; Vilalta-Perdomo 2005).

As indicated above, VSM considers five (sub-)systems and six communications channels to monitor and control the operations and to analyze the adaption processes within an organization. In this paper, these elements were the foundation to map SFSCs, through an approach that consisted of: (i) a system identification by modeling SFSCs current situation; (ii) a system diagnosis by studying what the SFSC is doing, and (iii) other requirements for viability by uncovering the practices followed by SFSCs (Rezaee et al. 2019). All these activities can be done with VSM through face-to-face workshops, where different stakeholders take part in conversations conducive to modeling the system-in-focus (Lowe et al. 2020). A series of in-depth interviews can also be used to support such modeling (Salinas-Navarro 2003; Vilalta-Perdomo 2005). However, the lockdowns that took place during the COVID-19 pandemic did not allow to follow any of such approaches; hence, a decision was taken to change the way these activities were conducted, and a series of modifications to the VSM graphical description were done and displayed through an online visual collaboration platform. A description of the requirements for the online mapping exercise and the adjustments done in VSM are presented in the following sections.

### Requirements for the Online Mapping Exercise on SFSCs

This research pilot involves a set of Mexican SFSCs, constituted by one hybrid collective (with attributes from cooperatives and community-supported agriculture) Colectivo Zacahuitzco, and four farmers’ markets: Feria de productores, Mercado Alternativo de Tlalpan, Mercado de las Cosas Verdes (Tianquiskilitl), and Tianguis Alternativo de Puebla; located in three Mexican big cities: Guadalajara (5 million inhabitants), Mexico City (21.8 million) and Puebla (3.2 million) (see Table 1). The selection of these SFSCs followed a purposeful sampling procedure to discover, understand, and learn as much as possible (Merriam 1998). Specifically, the aim was to cover the most common types of SFSCs documented in the literature: (a) farmers’ markets and (b) cooperatives (Michel-Villarreal et al. 2019). The selected SFSCs serve as illustrations of the most common types of SFSCs and display different characteristics in terms of their configuration, years of operation and size. The selection of Mexico as the fieldwork setting takes advantage of the authors’ familiarity with the context, but it also responds to the current bias in the geographical distribution of SFSCs research, as research on SFSCs in the global South remains scarce (Freidberg and Goldstein 2011; Michel-Villarreal et al. 2019).

**Table 1:** Participant organizations in the research pilot

Name	Description	Location
Colectivo Zacahuilco (Able to operate online during COVID)	A collective (hybrid SFSC combining aspects of coops and community-supported agriculture) constituted by producer and consumer families of organic-sustainable, urban-rural, food, health, and home products to barter.	Mexico City
Feria de Productores (Unable to operate online during COVID)	A community of producers and consumers that brings together ranchers and farms dedicated to food production and located in the municipalities surrounding the city of Guadalajara.	Guadalajara, Jalisco
Mercado Alternativo de Tlalpan (Able to operate online during COVID)	Autonomous self-sustaining space for coexistence where producers can market without intermediaries. It is an independent citizen initiative promoted by young inhabitants of the south of Mexico City.	Mexico City
Mercado de las Cosas Verdes Tianquiskilitl (Able to operate during COVID)	Sale of agroecological, local, seasonal, organic, and direct products from producers.	Mexico City
Tianguis Alternativo de Puebla (Able to operate online during COVID)	A social node (academics, consumers, producers and volunteers) who contribute to the fair exchange of knowledge, consumer alternatives, and healthy and sovereign food through meeting spaces and collaborative work based on sustainability and community building.	Puebla, Puebla

The national lockdown in Mexico affected these SFSCs in different ways; farmers’ markets saw the closure of their public venues and Colectivo Zacahuitzco (which operates in a private venue) had to implement social distancing measures in their physical store. An important enabler for the continuation of operations of four of these organizations was the adoption of online business models and home deliveries. The different experiences of the selected cases allowed us to investigate the organizational, logistical, and technological changes required for the digitalisation of SFSCs, as well as the associated challenges.

**Fig. 2:**
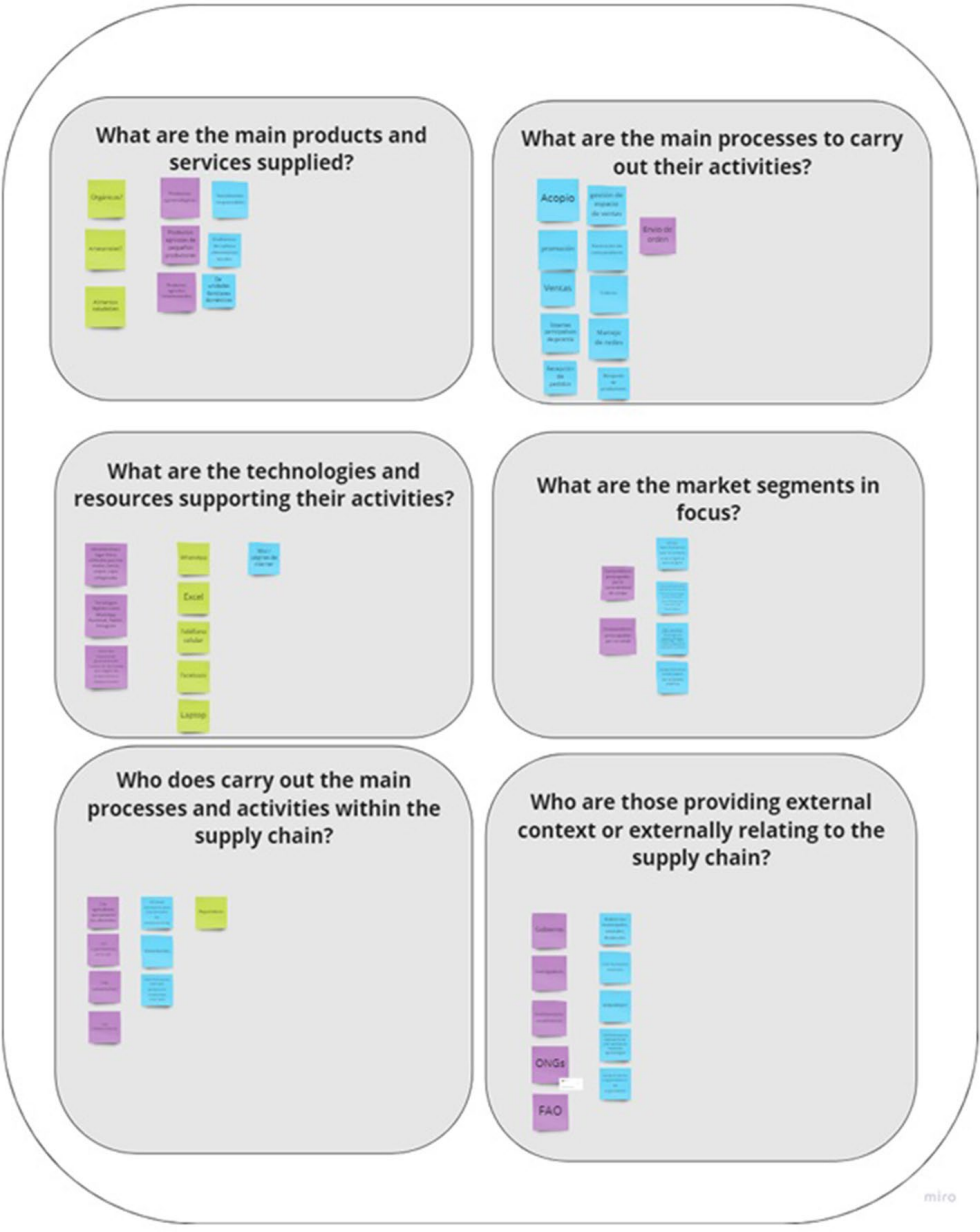
Miro© template to answer, ‘What is the system in focus?’

**Fig. 3:**
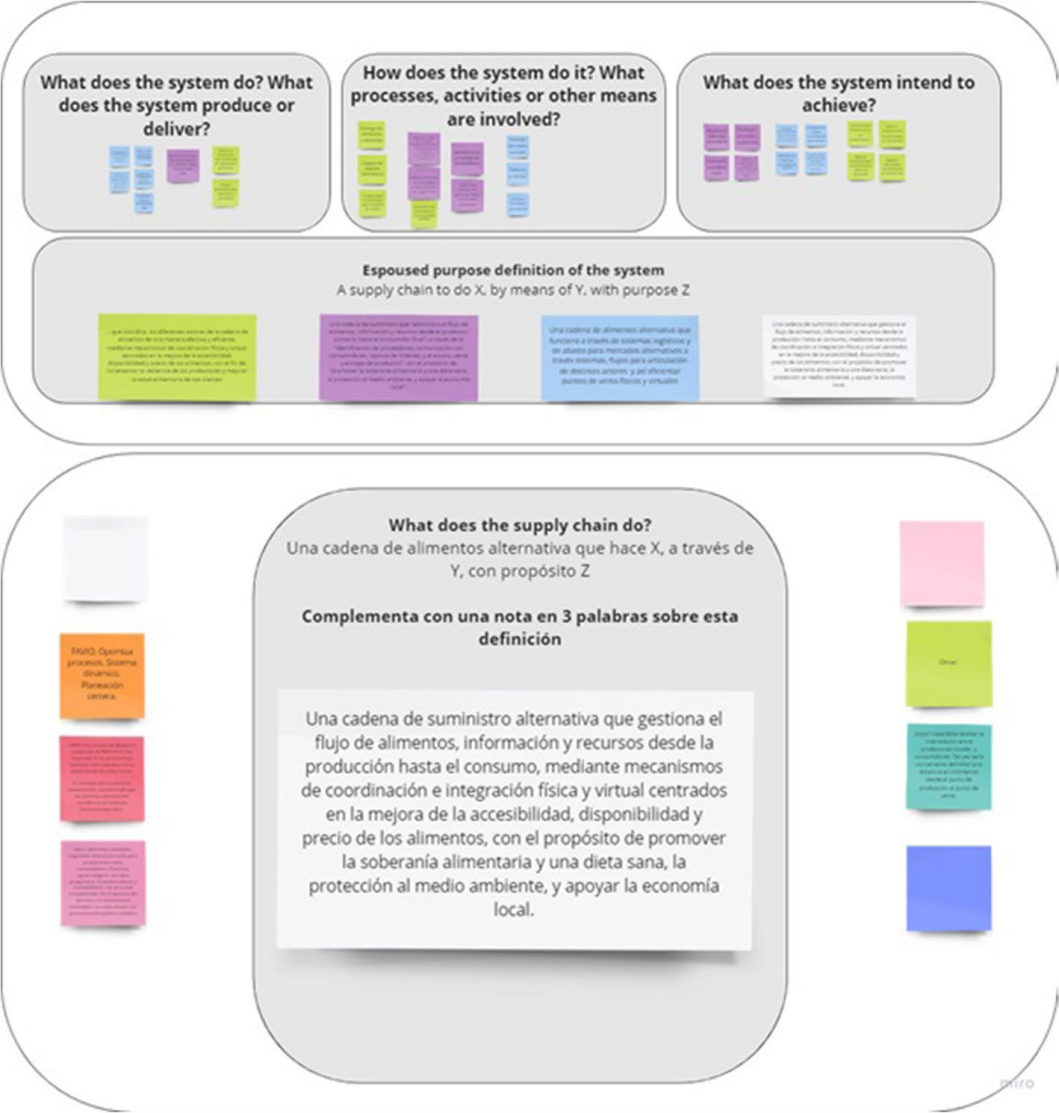
Miro© template to answer, ‘What does the system do?’

**Fig. 4:**
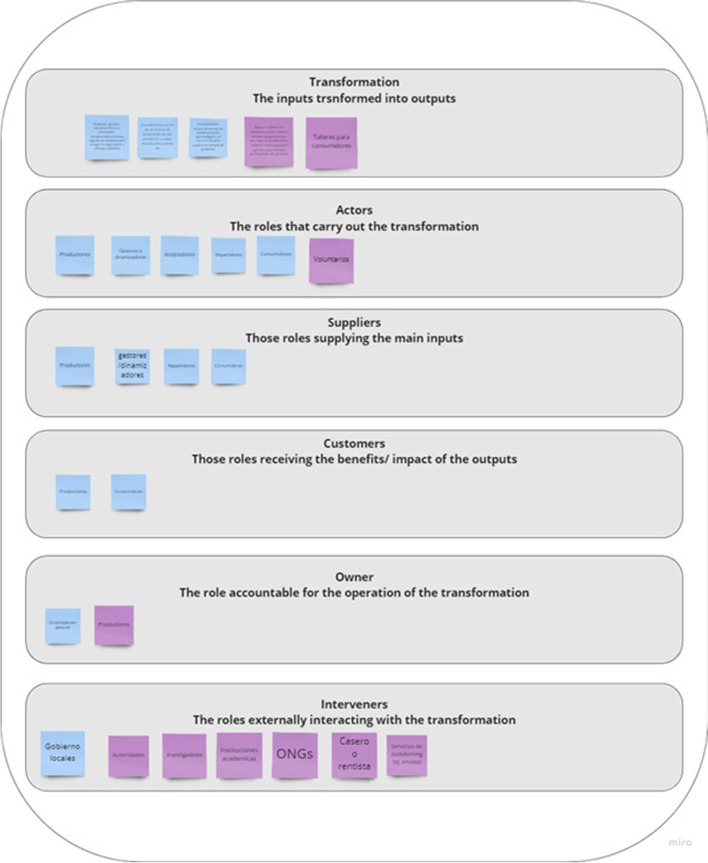
Miro© template to define the TASCOI

To build an initial understanding concerning the elements to be considered when mapping a SFSCs, 10 online semi-structured interviews (see Table 2) were conducted with key actors from two farmers markets (i.e., *Mercado Alternativo de Tlalpan* and *Tianguis Alternativo de Puebla*) to explore what the system in focus is and what the system does (see Figs. 2, 3 and 4). Furthermore, to explore their current operations and logistics processes and determine their technology needs, an online focus group was also held with managers from five SFSCs (see Table 1) to describe SFSCs subsystems in terms of the VSM systemic functions. Four of the five SFSCs were able to operate during the pandemic, as they had enough technological capabilities to conduct online operations. Only one of the five was unable to do so and closed during that period. A by-product of such focus group was testing the effectiveness of online VSM to map SFSCs.

**Table 2:** Questions to collect interviewees’ reflections

Relation	Questions:
Cohesion (3 questions)	• What activities do you carry out in the supply chain? • To whom do you relate in the supply chain to carry out your activities? • What resources/materials/products/information do they exchange in these interactions?
Performance (4 questions)	• Who benefits through your supply chain activities (internal and external)? • What activities or tasks do you carry out with the beneficiaries? • What do you exchange in your relationship? • What are the criteria/expectations of evaluation/satisfaction with the results?
Ownership (5 questions)	• From economic, social and/or environmental perspectives, why are you part of the supply chain? • What do you do to belong (integrate) to the supply chain? • What do you do to stay within the supply chain? (Responsibilities, duties or motivations) • Who do you relate to (agree with) to stay in the supply chain? • What do you exchange in this relationship?

### Online Focus Group: Graphic Description of VSM Using the Miro© Platform

Miro© is an online visual collaboration platform that “empowers remote, in-office, and hybrid teams to communicate and collaborate across formats, tools, channels, and timezones [sic] — without the constraints of physical location, meeting space, and whiteboards” (Miro n.d.). Miro© consists of a web-based white canvas where several individuals can collaborate simultaneously for brainstorming. The rationale behind its selection is the simplicity of the system, as it can be explained online in only a few minutes, and participants do not need to sign up to the platform. To participate in a meeting supported by Miro©, only requires clicking on a link sent by the organizers in an email invitation.

The first step of VSM helps to identify what the system-in-focus (SFSC) is, by modeling its current situation. Such modeling involved the identification of: (i) main products and services provided, (ii) the main processes, (iii) technologies and resources used, (iv) market segments covered, (v) main actors in the SFSCs, and (vi) external links. These questions were encapsulated in Miro© through a series of the boxes, where the workshop participants introduced their answers, by means of “digital” post-it notes (see Fig. 2).

The second step of the VSM mapping concerns diagnosing the system-in-focus (SFSC), by uncovering what the SFSC is doing. This is usually solved by ‘naming the system’, through describing the system’s ‘root definition’ by means of the CATWOE (Checkland 1981), or identifying the relevant organizational systems, using the TASCOI (Espejo et al. 1999). It is relevant to notice that CATWOE and TASCOI are used to identify the transformation implied by the purpose and the relevant stakeholders (Harwood 2021). The outcome of *naming the system* is a definition of what SFSCs consider is said to be doing. Conversely, VSM centers the attention on what the system does, rather than what it says it does (Beer 2002). Accordingly, in this step, in addition to do an external description of the system-in-focus, three reflective questions were proposed: (i) What does the organization do? (*cohesion*) (ii) How does the organization do it? (*performance*) and (iii) Why does the organization do it? With what purpose? (*ownership*) (see Figs. 2, 3 and 4).

These three questions were decomposed for allowing a detailed reflection on which were the relations among the different constitutive elements of the SFSC. This resulted in 12 questions (see Table 2) that were used in semi-structured interviews to understand the different views of what a SFSC does depending on the role that the interviewed play.

**Fig. 5:**
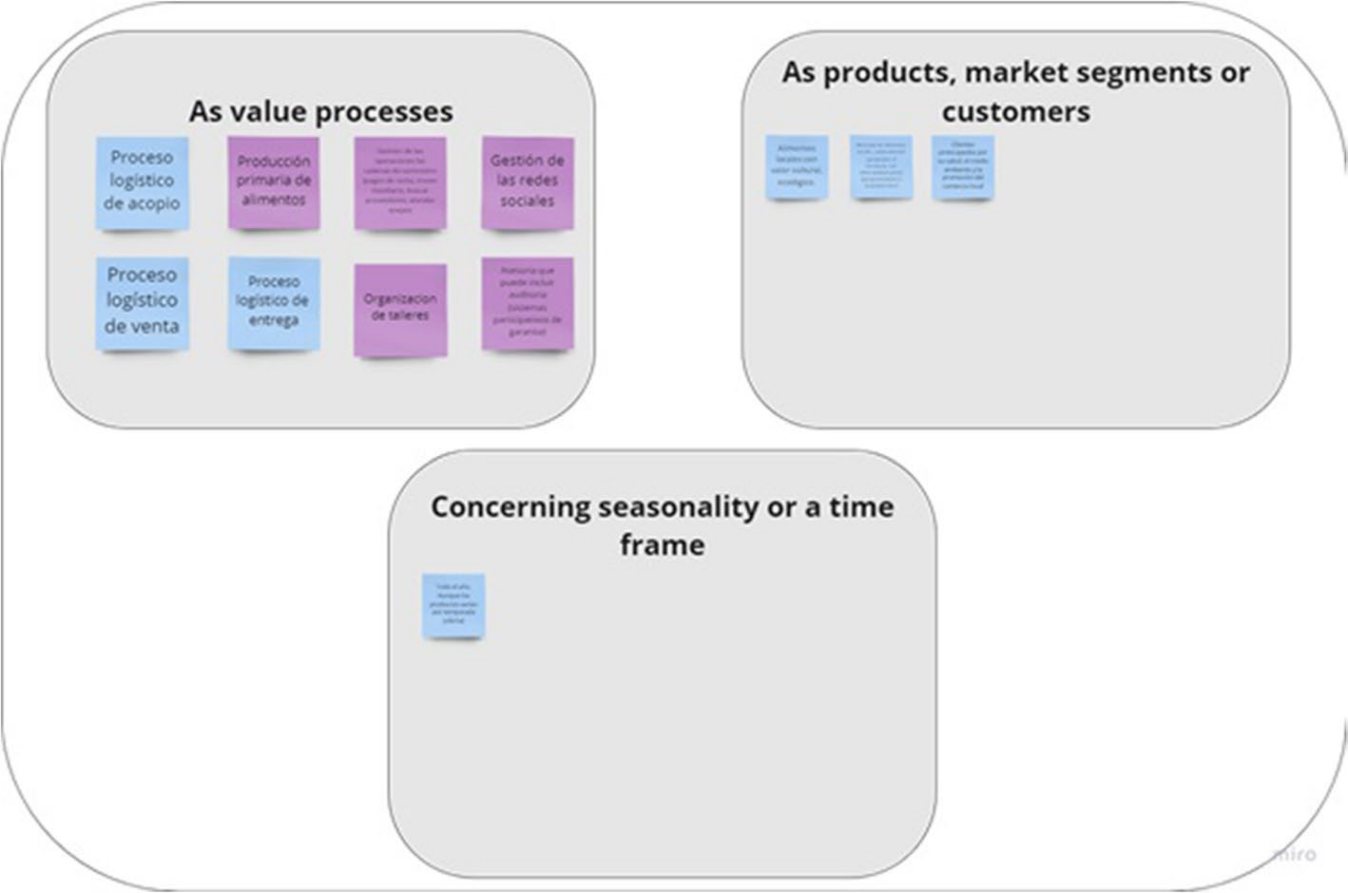
Miro© template to identify the subsystems (structural units)

The third step consists of a collective reflection on other requirements for achieving viability; this considers the structural units that constitute the SFSC. In this step, an online workshop was conducted for participants to discuss how SFSCs subsystems or structural units are organized in terms of (i) value creation processes, (ii) as products, markets or customers, and/or (iii) in terms of time (throughout the year, seasonal, or other temporal patterns (see Fig. 5).

**Fig. 6:**
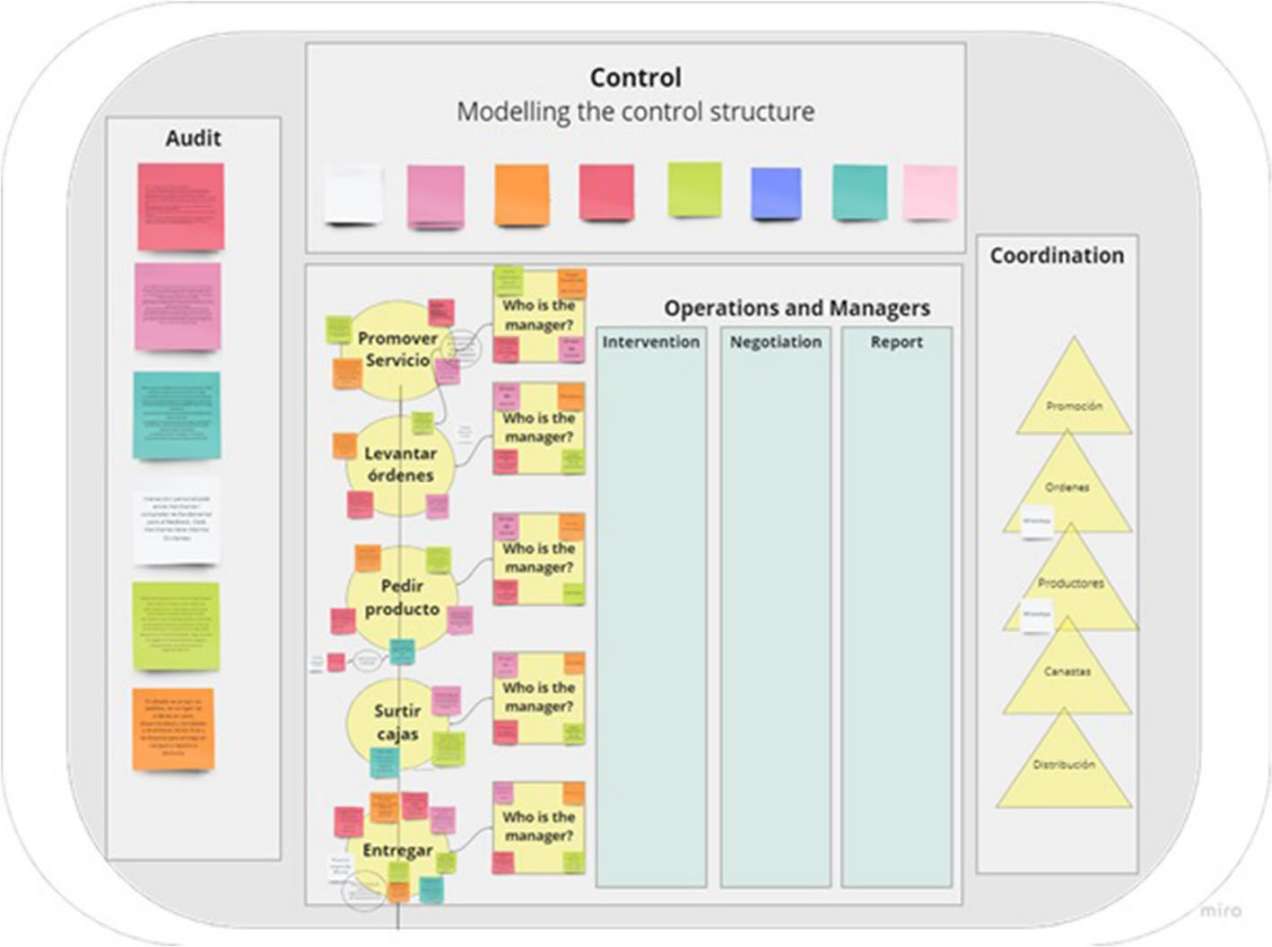
Miro© template to answer, ‘How do activities are carried out?’

In this step participants could identify VSM’s systems One (Implementation); Two (Coordination), Three (Control) and 3* (Audit), and the six vertical channels (Intervention, Resource Bargain, Accountability, Operational Axis, Co-ordination, and Audit see Fig. 6). Figure 6 was built based on the traditional VSM graphical description (see Fig. 1), but boxes were added to allow participants to include their views through post-its.

However, as discussed more in detail below, during the focus group participants were mainly focused on describing System 1 (operations) and System 3* (audit) and some elements of System 2 (coordination).

### Data Collection and Analysis

As indicated above, this study adopted semi-structured interviews as one of the methods to collect data because these facilitate the collection of rich data related to participants’ views (Saunders et al. 2016), whilst providing the required degree of structure and flexibility (Merriam 1998), and increasing reliability by ensuring that the data collection procedures can be replicated more easily (Yin 2014). Online interviews were administered using video-conferencing platforms, which allowed to reach participants that were not otherwise accessible due to the COVID-19 pandemic. The semi-structured interview template can be seen in Appendix Table 4. Data were obtained from ten interviewees were interviewed (i.e., customers, producers, managers, and volunteers, from Mercado Alternativo de Tlalpan and Tianguis Alternativo de Puebla). The average interview length was 40 min. They were audio recorded and later transcribed for analysis. A total of ten transcripts containing around 60,000 words were analysed. The data were collected during the first semester of 2021. SFSCs usually involve three main actors: producer (or supplier), organizer, and consumer (Michel-Villarreal et al. 2021). However, digitalized SFSCs seem to involve additional key roles, such as pickers and couriers. Therefore, the selection of interviewees was based on diversity and their ability to provide different perspectives to reduce potential interview bias, prioritising personal contributions to the development of insight and understanding of the phenomenon (Merrian 1998, 83).

The semi-structured interviews were followed by an online focus group (also referred to as ‘workshop’ in this paper) using Miro©. A focus group is a group interview that focuses on a particular issue “by encouraging discussion among participants and the sharing of perceptions in an open and tolerant environment” (Saunders et al. 2016, 420). Online focus groups provide several advantages, including full and equitable participation of hard-to-reach populations and cost-effectiveness (Tran et al. 2021). The design of such focus group followed the principles proposed by Ramírez and Fernández (2005): (i) to engage the relevant stakeholders in a process of learning and negotiation; (ii) to ensure that a balanced group of stakeholders was brought together; (iii) to demonstrate, through structured, practical exercises, that the underlying causes of conflict are often the result of stakeholders not being able to negotiate diverse interests; (iv) to unravel conflicts by having stakeholders explain their world-views and perspectives using visual tools, and (v) to provide stakeholders with a summary of workshop deliberations that would allow them to replicate and improve the process with the constituencies that they represent. A total of five participants from five SFSCs and three moderators were involved. The length of the focus group was 1 h 52 m. It was video recorded and later transcribed *verbatim* resulting in a word count of 14,976.

A codebook of theoretically-derived codes based on the VSM was developed to increase reliability (see Appendix Table 5 for an excerpt). The codebook included a set of structural codes for each question within the interview template to guide the coding process (Ryan and Bernard 2003). Subsequently, a more inductive process of coding was carried out based on a six-phase thematic analysis approach (Braun and Clarke 2006). Transcriptions from semi-structured interviews and the focus group were imported into the NVivo software and analysed separately. Phase 1 required familiarization with the data and established a preliminary understanding of possible emergent patterns. Phase 2 called for the identification of initial codes across the data. Phase 3 involved categorizing the identified codes into emergent themes. Here, consideration was given to how different codes may fall under a wider theme. In phase 4, identified codes and themes were refined. This step involved reviewing whether the codes within each theme were consistent and form a coherent pattern. In phase five, names were assigned to the overarching themes based on the main aspects of the data that they represented. Lastly, phase 6 involved the writing up of individual case reports. Appendix Fig. 8 illustrates the overarching themes and codes identified via NVivo.

Several criteria consistent with qualitative research were used to ensure the goodness or quality of the research (Lincoln and Guba 1985; Moon et al. 2016) (see Table 3 below).

**Table 3:** Suggestions to achieve research quality and rigor

	Research phase		
Criteria	Design	Data collection	Data analysis
Dependability	Development of codebook based on theoretical framework (see excerpt in Appendix Table 5).	Use of semi-structured interviews and the development of a layout for the focus group to increase replicability.	Independent coding and agreement by two researchers.
Credibility	Guided by theoretical constructs (i.e., VSM).	Selection of participants with different perspectives/roles.	Member validation of interview findings with a focus group.
Transferability	Maximizing the diversity of SFSCs included in the research.	Use of different data collection methods (i.e., semi-structured interviews and focus groups), to check the consistency of the findings where possible.	Corroboration of findings from different SFSCs and data collection methods.

## Findings

A series of findings were collected from running the online VSM workshop and the semi-structured interviews. These can be divided in two categories: (i) findings concerning the SFSC mapping, and (ii) findings on the use of VSM via online platforms.

### Findings Concerning the SFSC Mapping

SAFeNET considers that local food products and their cooking are part of cultural identity, hence the interest to preserve, disseminate and value local food. SAFeNET paid special attention to how SFSCs contribute to preserving cultural processes and family dynamics around production, processing, exchange, and consumption in the daily dynamics of small producers. SAFeNET also helped to observe how women, young people, and children participate in the preservation of identity and culture through food. Furthermore, SAFeNET investigated the impact of SFSCs on the preservation of cultural manifestations. Farmers’ Markets that participated in this collective research showed that pre-Hispanic food and cooking styles were encouraged in conversations between producers and consumers. The Farmers’ Markets show other cultural aspects where families were integrated in such a way that the Farmers’ Markets became more than a commercial place for exchange. For instance, some of the Farmers’ Markets became communities that organized events where children played, and adult customers learned different skills by visiting some of the food producers’ facilities. Some of these events involve sharing cooking skills and recipes, providing nutritional facts, and recommendations on how to grow their vegetables at home, such as varieties of chilies; they also provide instances of exchanges of good and bad experiences in the manipulation and use of local produce, and cooking procedures.

As indicated above, 10 semi-structured interviews were conducted to build an initial understanding concerning the elements to be considered when mapping a SFSC (see Table 2). Different members (i.e., customers, producers, managers, and volunteers) of two farmers’ markets (i.e., *Mercado Alternativo de Tlalpan* and *Tianguis Alternativo de Puebla*) were interviewed. These semi-structured interviews aided to build an initial collective understanding or definition of what a SFSC is, which was later tested in an online focus group where organizers from five SFSCs participated. The semi-structured interviews showed the relevance of SFSCs for the conservation of local food ecosystems, the diversification of distribution processes and local sales, the maintenance of healthy food choices, and their support for local family economies (rural and urban). The semi-structured interviews also helped to develop a preliminary list of activities required to run a SFSC. This list was also tested in the online workshop, and it includes the following: (i) to promote the service; (ii) to collect orders; (iii) to inform producers; (iv) to process orders, and (vi) to deliver orders.

The focus group outcomes were, first, a collective and validated definition of what a SFSC is. The initial description (naming the system) that emerged from the interviews was adjusted to consider additional elements, such as: (i) the importance of accurate planning; (ii) the dynamic nature of how SFSCs operate; (iii) the importance of territory and distance between consumer and producer; (iv) SFSCs as a political movement, and (iv) their importance in terms of food security and sovereignty. As an outcome of the workshop, a SFSC was collectively described as:

An alternative supply chain that manages the flow of food, information, and resources from production to consumption, through physical and virtual coordination and integration mechanisms focused on improving the accessibility, availability, and price of food, with the purpose of promoting food sovereignty and healthy diets, protect the environment, and support the local economy.

The second outcome concerned the roles that people involved in digitalised SFSCs play. These roles, identified during the semi-structured interviews, were confirmed in the focus group. Accordingly, digitalized SFSCs operations require at least the following roles: producers, organizers (*coordinadores*), pickers (*marchantes*), couriers, and consumers.

**Fig. 7:**
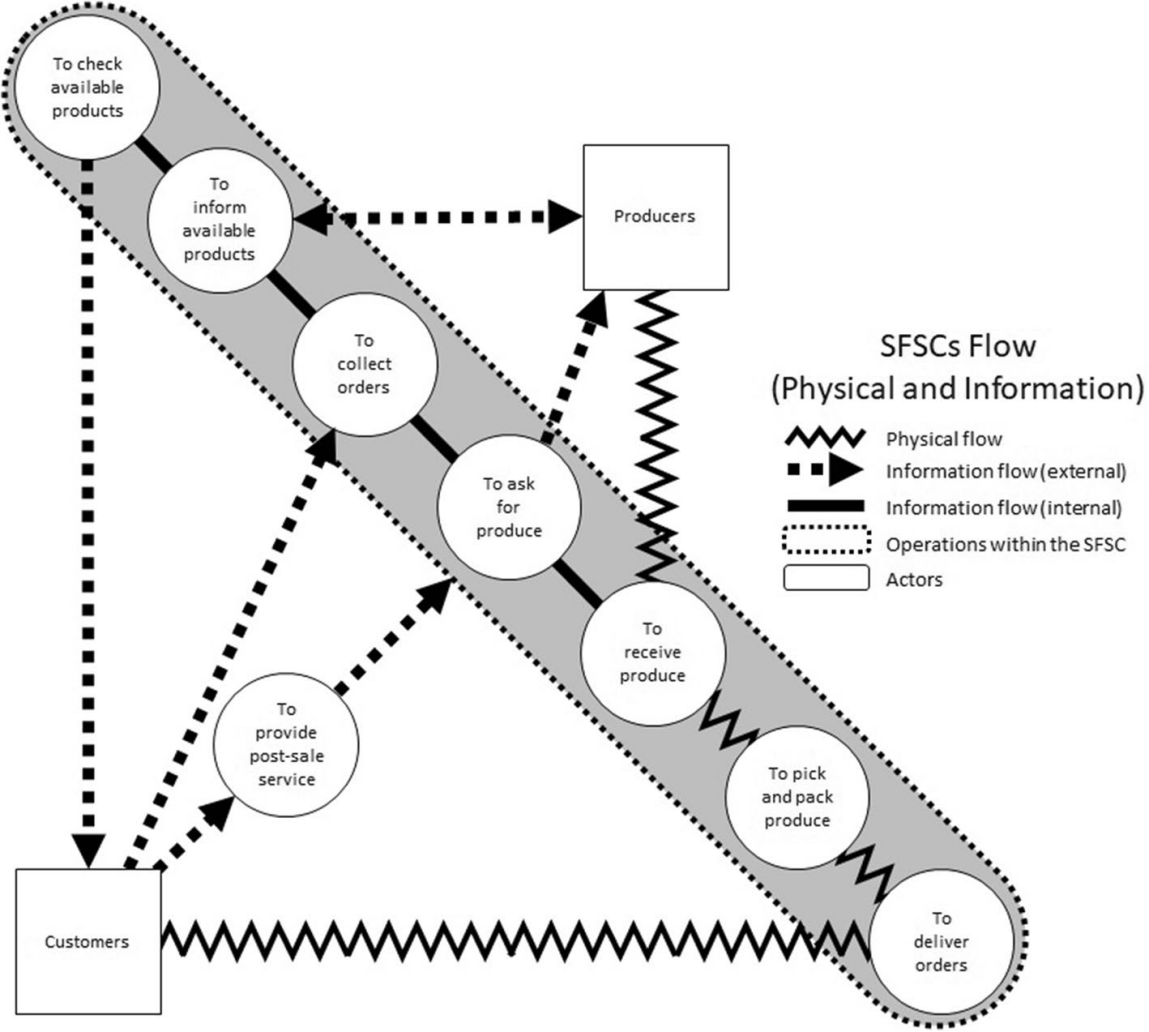
Schematic of the short food supply chain flow (physical and informational)

The third outcome refers to the activities required when running digitalized SFSCs. Even though all SFSCs agreed on the same steps of the process as shown in Fig. 7, each approach varied slightly. Each step and the associated variations are explained below:

- Step 1 – To check product availability: This step involves organizers and producers checking product availability (at the farm level). Due to the seasonality of agricultural produce, product availability needs to be updated on a weekly basis to reflect an accurate inventory.
- Step 2 – To communicate product availability: Consumers are notified of the product availability in advance of order collection. Strategies for the communication of product availability varied but most initiatives made use of WhatsApp groups, Facebook, own websites and/or e-mail. The responsibility for this step usually falls on organizers.
- Step 3 – To collect orders: This step involves consumers creating their orders and SFSCs (often organizers) collecting those orders. For this step, SFSCs have adopted different approaches depending on the digital technologies that they have available. For instance, *Tianguis Alternativo de Puebla* has already developed a website that automatizes the collection and processing of orders to some extent. The website allows consumers to add products to their carts and pay online. The system also sends order confirmations via email after consumers checkout. However, *Mercado Alternativo de Tlalpan* and *Mercado de las Cosas Verdes* do not own a website, so the process is more manual. Product availability is communicated via an Excel file that consumers download from either websites, email, or WhatsApp, modify and use as an order form, and send back when completed.
- Step 4 – To ask for produce: This step involves the aggregation of all weekly orders (usually by organizers). This involves compiling all orders and disaggregating by producer using Excel. This way, each producer is notified (usually via WhatsApp) of the produce that they need to deliver.
- Step 5 – To receive produce: SFSCs receive the produce directly from producers in a physical venue on Saturdays or Sundays. Producers are responsible for transportation to said venue. Organizers and pickers work together to receive the produce and check that each producer delivers what was requested in advance. They also adjust consumers’ orders if there have been changes due to product availability at the farm level or quality loss.
- Step 6 – To pick and pack produce: Organizers or pickers sort out the produce delivered by producers in designated tables. Then, they select items for the different consumers’ orders and store them in assigned boxes or baskets. This step may also require the use of some additional packaging (i.e., paper or plastic bags) and labelling.
- Step 7 – To deliver orders: The responsibility for routing, scheduling, and delivery of products to final consumers varies across the different SFSCs. In some instances, this step is owned by organizers and pickers, and for others the activities are carried out by an outsourced courier (e.g., Uber, Bicientregas, etc.). After the order arrival, received orders are collected and checked by the consumer. This step may also include the processing of a payment or product return. Additionally, consumers have the option of collecting their orders from a physical venue, which does not require routing, scheduling, and transportation. Orders collected by consumers are usually handled by organisers or pickers.
- Step 8 – To provide post-sale service: At the end of the process, organizers are responsible for verifying and disaggregating consumer payments and re-distributing disaggregated payments to producers. This step may also include handling returns and consumers’ complaints, as well as VAT payments.

Lastly, other findings from the interviews and the workshop concerning the mapping of digitalized SFSCs, including challenges, are summarized below:

- Digital technologies in use to communicate among different members were email, Google Meet, WhatsApp, YouTube, Facebook and Zoom.
- During COVID-19, *WhatsApp* chats were considered the best tool to enhance visibility and facilitate the flow of information along the supply chain. For example, SFSCs supported their online operations mainly by using *WhatsApp* to receive orders from consumers and *Excel* to compile and consolidate orders.
- Online SFSCs comprise five key steps: promote the service; receive and process online orders; request goods; receive, pick, and pack goods; and deliver orders, plus a series of supportive steps, such as: confirm what to offer, payment to producers, post-sale services, and general administration (e.g., tax declaration, and producers’ internal audits and accreditations).
- There was an extended agreement regarding the need for more sophisticated and ad hoc technologies such as mobile apps and websites, to improve the efficiency of the online operations.
- Some of the main challenges when digitalizing SFSCs include the seasonality of produce which demands updating inventories on a weekly basis, the high costs associated with the development of online platforms and apps, and the high commissions and delivery fees asked by existing online food ordering and delivery platforms.

### Findings Concerning the Pertinence of Online VSM.

SAFeNET’s main contribution concerning Systems Thinking practice and the Viable System Model (Beer 1972) was the development of an online collaborative conversational tool for self-reflection and collective learning in social contexts, aligning the organization of people with their purposes and views. That is, the tool provides a framework that can help people talk about their communications and interactions with other actors with a sense of their own purpose, identity, and structure, about their needs to operate and coordinate better, and the challenges of their interactions with other stakeholders, and their environmental challenges. In this respect, the most relevant output was the development of a visual interactive tool, based on the Viable System Model, to support the efforts of collectives strengthening their autonomy and viability. This tool, supported on the Miro© platform and tested in an online focus group, allowed mapping the operations associated with the logistics of the digitalized SFSCs. This tool was effective to identify operational patterns, which can be automated in computer-based software.

Additional findings concerning the online use of the VSM are as follows:

- First, it was confirmed that the VSM can be implemented online. However, there is the need to use an online platform that allows participants to replicate face-to-face workshops; for instance, supporting visual interactive communication (e.g., Miro©).
- How to engage? Online workshops require a well-developed structure. Participants need clear instructions for what is expected, and sufficient time to reflect and write their comments and experiences. Plan B is needed for participants who are lost in the process.
- Effectiveness and efficiency? The process is slower, but writing allows participants to develop a more in-depth reflection. However, it is recommended to record online workshops to avoid loss of information that has not been captured in post-it notes by participants. During our workshop, most people engaged with writing via post-it notes, but a few found it difficult and preferred to answer the questions verbally.
- The VSM was used as an online conversational framework to guide the exploration of people’s views and the understanding of their situation rather than focusing on the graphical representation of the VSM or the systemic language of the researchers.

## Conclusions. Impacts in Practice and Research

The initial phase of SAFeNET reported in this paper strengthened an incipient UK/Mexican research partnership focused on designing better decision-making for logistics operations within SFSCs. Conversations began in 2019 and gained further relevance when COVID-19 impacted food systems worldwide. During 2020, data collection and webinars maintained the flow of ideas and concerns, but financial support was required to progress this collaboration. In 2021, funding from the Global Challenges Research Fund (GCRF) quality-related research (QR) was received through the Aston University allocation.

SAFeNET’s main academic contribution from this first step was to test the appropriateness to use the Viable System Model (Beer 1972) as an online collaborative conversational tool. Our proposed framework provided space for self-reflection and collective learning in social contexts, aligning the organization of people with their purposes and views. Accordingly, the most relevant academic output was the development of a visual interactive tool, based on the Viable System Model. This tool, supported on the Miro© platform and tested in an online workshop, supported the mapping of operations associated with the logistics of the different digitalised SFSCs. This tool allowed the identification of operational patterns, which might be automated in computer-based software in the future.

Furthermore, members from the five SFSCs participating in this research contributed to discussions concerning how their resilience was tested during the COVID-19 period and how it could be improved. Accordingly, organizational, logistical, and technological processes for collective online selling were investigated. The outcomes included the recognition of economic, informational, logistical, organizational, and technological elements that could increase their resilience. The main benefit has been that as all the SFSCs involved in this research operate in similar ways, and operational patterns were observed, there is a possibility to automate their operations through digital web-based technologies. This supports SAFeNET hypothesis that digital decision-making tools of general application can and should be developed to support the digitalisation of SFSCs logistical operations.

However, there were important differences associated with technological abilities. As indicated above, four SFSCs were able to develop online selling systems, but one was not and had to close their operations during the COVID-19 pandemic. This had a negative impact on some farmers’ livelihoods as they were left without a selling outlet. The focus group’s participants reflected upon their procedures and operations following the VSM elements, sharing knowledge and experiences, identifying necessary improvements, and confirming which pre-COVID selling procedures were resilient.

### Impact on Research and Practice

The mapping of digitalized SFSCs described in this study contributes towards filling a gap in current literature of SFSCs and could serve as guidance for practitioners wanting to implement similar online selling systems. Thus, SAFeNET first step succeeded in identifying the elements required to design an efficient and effective digital order fulfillment app that can better support the continuity of SFSCs operations. Future steps of SAFeNET will focus on the development and testing of such a web-based digital order fulfillment device.

The first step of SAFeNET reported in this paper helped to recognize that online VSM opens the opportunity to increase participation. This step also confirms that systems thinking interventions can be done through online tools. Some benefits from such online interactions is that participants in different locations can interact through collective knowledge-building exercises. This implies expanding the role of participants from being objects to be observed, to becoming subjects that reflect and learn from what they are observing. In this context, participants do more than just answer close questions (surveys) or provide their views in a less structured approach (interviews); participants may become co-researchers. However, some challenges associated with distance participatory methods need to be considered. Hall et al. (2021) suggest considering the following:

- Access to networks and devices. Even though physical distances fade in a virtual world, computer-based technological barriers may raise, particularly in marginalized/rural areas. Other aspects associated with technology challenges may involve information technology (IT) literacy and the cost of data.
- Equal opportunities for engagement. Different situations may jeopardize or at least limit participation through online technologies, such as disabilities. In this respect, there are also issues concerning lack of control, as participants cannot be closely supervised and may lose interest in what is going on. This requires establishing means of virtual communication that allow spontaneous interactions and create a shared space for collaborative knowledge learning.
- Finally, there are aspects related to privacy, confidentiality, and data collection policies, depending on the platform used.

In summary, when planning an online focus group that includes the use of a systems thinking approach, we need to consider that some time is required for participants to understand the tool (e.g., Miro©). Furthermore, it involves a slower process that limits the amount of information captured, but, at the same time, such higher quality in responses, and allows for higher participation.

Finally, it is important not to forget that ways to maintain attention and engagement must be put in place. The VSM mapping of the SFSCs operations reported in the paper was constrained to one session of 2 h, where the exploration was focused on identifying: (i) the operational flow, what are the activities done in online selling; and (ii) concerning the control subsystems in place. A brief conversation on (iii) the coordination mechanisms established to maintain consistency among the operations; (iv) the vertical channels (i.e., intervention, resource bargain, and accountability) also took place, but no great detail was achieved.

### Future Research

Some limitations have been identified concerning online VSM exercises. First, face-to-face workshops can consist of long sessions, interrupted by short breaks, where VSM can be properly developed, but this is not possible through an online platform, as the attention span is more limited. Probably, further explorations could be done by employing additional focus groups, but for the aim of the first step of SAFeNET this was not required.

Another question that deserves further investigation is to explore how to increase the length of online VSM workshops. In the focus group reported, we tried to identify ways to increase participants’ perceived value in exchange for being involved. On this occasion the research team focused the attention on two elements: (i) to confirm that what they do is like what the rest do; patterns in their operations were recognized and made explicit; (ii) to identify areas of opportunity, online experiences were exchanged, and limitations discussed. However, other possible elements might be explored, such as adding breaks, eliminating distractions, or building a game.

In summary, it seems worth doing further research concerning the development of online versions for other Systems Thinking methodologies (e.g., SD, SSM), but detailed design and testing is required. Additionally, further actions and research should be carried out to support SFSCs’ organization in informational and operational terms. The development of more effective business-to-consumer (B2C) interfaces for supporting online selling requires more efficient business-to-business (B2B) interfaces between SFSCs and food producers.

The research leading to these results received funding from UK Research and Innovation, under the Grant: ASTON GCRF QR 2020-21.

## Figures and Tables

**Fig. 8:**
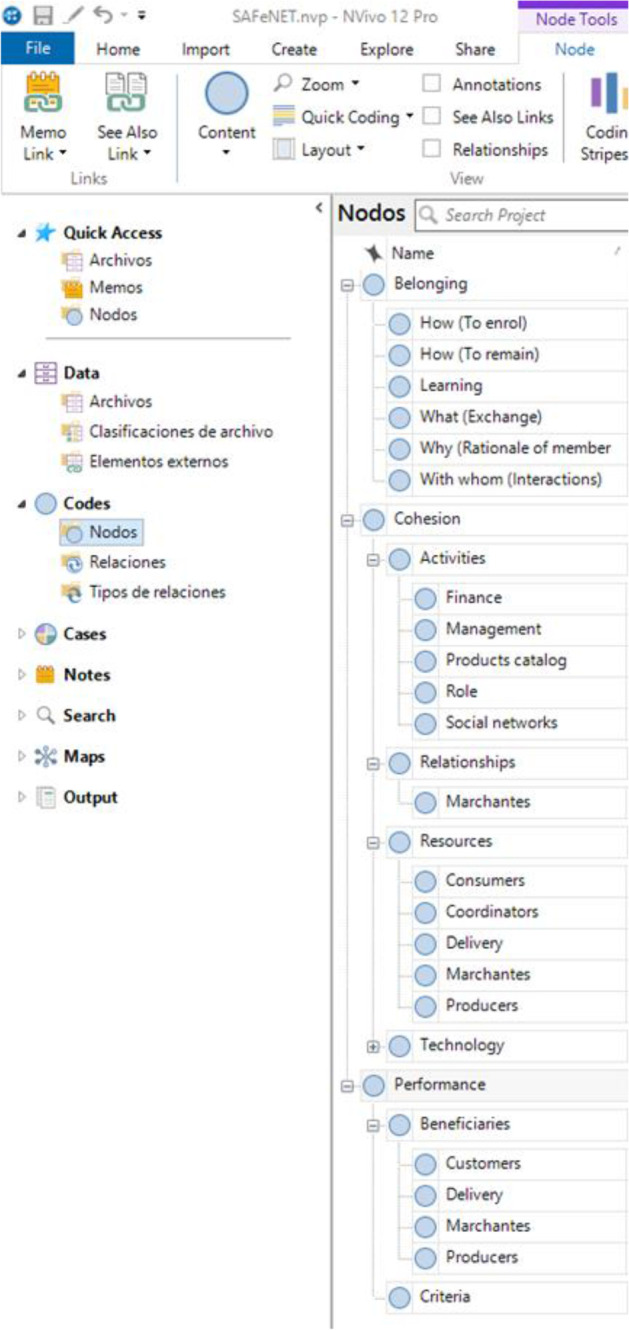
Coding process using NVivo

## Data Availability

The data sets generated and analysed during the current study are not publicly available due to their nature. They involve the transcript of a workshop where the participants were recorded but preferred to remain anonymous. The transcript may still be available if it is a reasonable request.

## Appendix

Table 4, 5; Fig. 8

**Table 4:** Semi-structured interview template

Relation	Questions:
Cohesion (3 questions)	• What activities do you carry out in the supply chain? • To whom do you relate in the supply chain to carry out your activities? • What resources/materials/products/information do they exchange in these interactions?
Performance (4 questions)	• Who benefits through your supply chain activities (internal and external)? • What activities or tasks do you carry out with the beneficiaries? • What do you exchange in your relationship? • What are the criteria/expectations of evaluation/satisfaction with the results?
Ownership (5 questions)	• From economic, social and/or environmental perspectives, why are you part of the supply chain? • What do you do to belong (integrate) to the supply chain? • What do you do to stay within the supply chain? (Responsibilities, duties or motivations) • Who do you relate to (agree with) to stay in the supply chain? • What do you exchange in this relationship?

**Table 5:** Codebook based on structural coding (excerpt)

Interview Topic	Q#	Structural Code	Structural Code Definition
Activities	1	Cohesion_Activities	**Brief Definition**: Discussion of the activities that participants carry out within the SFSC
			**Full Definition**: Participants’ views regarding the activities that they are involved with as part of the role within the SFSC. Interview Guide question: • What activities do you carry out in the supply chain?
			**When to Use**: Use this code to capture opinions regarding the activities carried out by participants that are in response to Question 1, and all associated probes.
Relationships	2	Cohesion_Relationships	**Brief Definition**: Discussion of participants’ interactions with other members of the SFSC.
			**Full Definition**: Participant views regarding the relationships that they have stablished with other members of the SFSC in order to carry out their activities. Interview Guide question: • To whom do you relate in the supply chain to carry out your activities?
			**When to Use**: Use this code to capture opinions regarding the relationships of participants that are in response to Question 2, and all associated probes.
